# Sister Mary Joseph's Nodule Revealing Klatskin's Cholangiocarcinoma at the Yalgado Ouedraogo University Hospital: Case Report and Literature Review

**DOI:** 10.1002/ccr3.71337

**Published:** 2025-10-22

**Authors:** Lawagoulé Joseph Emile Ky, Eric Hervé Mourfou, Abdoul Fatao Maïga, Daouda Sangaré, Damien Marcel Traoré, Saïdou Santi, Madina Koanda, Alimata Sanfo, Delphine Napon/Zongo, Aboubacar Coulibaly, Kounpiélimé Sosthène Somda, Arsène Roger Sombié

**Affiliations:** ^1^ Hepato‐Gastroenterology Department Yalgado Ouedraogo University Hospital Ouagadougou Burkina Faso; ^2^ Pathological Anatomy and Cytology Department Yalgado Ouedraogo University Hospital Ouagadougou Burkina Faso; ^3^ General Surgery Department Yalgado Ouedraogo University Hospital Ouagadougou Burkina Faso; ^4^ Radiology Department Yalgado Ouedraogo University Hospital Ouagadougou Burkina Faso; ^5^ Dermatology and Venereology Department Yalgado Ouedraogo University Hospital Ouagadougou Burkina Faso; ^6^ Hepato‐Gastroenterology Department Sourô Sanou University Hospital Bobo‐Dioulasso Burkina Faso

**Keywords:** Africa, cholangiocarcinoma, Klatskin tumor, Sister Mary Joseph nodule, umbilical skin metastases

## Abstract

Umbilical cutaneous metastases of abdomino‐pelvic tumors are rare. They are known as Sister Mary Joseph nodules. The presence of this nodule indicates an advanced cancerous lesion and is associated with a poor prognosis. Because of its rarity, the Sister Mary Joseph nodule can go unnoticed. Its discovery should prompt a skin biopsy and abdomino‐pelvic cross‐sectional imaging. The occurrence of a Sister Mary Joseph nodule secondary to cholangiocarcinoma is rare. The aim of our work was to present a case of umbilical cutaneous metastasis revealing a Klatskin's cholangiocarcinoma in the Hepato‐gastroenterology department of the Yalgado Ouedraogo University Hospital (CHU YO).

**Summary**

- This is a case report for a patient with an umbilical metastasis, Sister Mary Joseph's nodule, from a hilar cholangiocarcinoma.
- Umbilical metastasis from cholangiocarcinoma is rare.
- The presence of a Sister Mary Joseph's nodule generally indicates poor prognosis. In this case, the patient died 2 months after diagnosis.

## 1 Introduction

Cutaneous umbilical metastases of abdominopelvic tumors are rare. Indeed, they are observed in around 1%–3% of abdomino‐pelvic neoplasias [1, 2]. They are known as Sister Mary Joseph nodules. In 1854 and 1860, umbilical metastases had already been described by Baluff and Nelaton, respectively. However, it was in 1928 that William James Mayo, son of William Worrall Mayo, founder of the Mayo Clinic, published the best‐known description of carcinoma metastases in this location, known as the “pants‐button umbilicus”. This phenomenon was reportedly noticed by William Worrall Mayo's nurse, Sister Mary Joseph. The term “Sister Mary Joseph nodule” was first used in 1949 by Hamilton Bailey, in his textbook “Physical Signs in Clinical Surgery” [3]. It is a discreet sign that often goes unnoticed due to its rarity. In fact, it is a sign of advanced primary neoplasia and poses problems of etiological and differential diagnosis, especially in our working environment, which is marked by insufficient technical resources. Although umbilical cutaneous metastases are rare, the occurrence of a cholangiocarcinoma metastasis at this site is even more so [4, 5, 6]. To our knowledge, the literature does not report any cases of a Sister Mary Joseph nodule secondary to cholangiocarcinoma in sub‐Saharan Africa, although it cannot be excluded that such cases may exist but have not been published [6]. The aim of our work was to present a case of an umbilical cutaneous metastasis revealing a Klatskin's cholangiocarcinoma in the Hepato‐gastroenterology department of the Yalgado Ouedraogo University Hospital (CHU YO).

## 2 Case Presentation

### 2.1 Clinical Examination

This was a 45‐year‐old patient hospitalized for epigastric pain associated with clinical cholestasis. He was a chronic carrier of the hepatitis B virus and did not consume alcohol or tobacco. The symptomatology began 4 months ago with the onset of burning abdominal pain, predominantly in the epigastric area, worsened with food. A month later, this pain was accompanied by a painful, pruritic umbilical tumefaction and a clinical cholestasis syndrome. Given the deterioration in his condition, the patient consulted the hepato‐gastroenterology department of the Yalgado Ouedraogo University Hospital.

**FIGURE 1:**
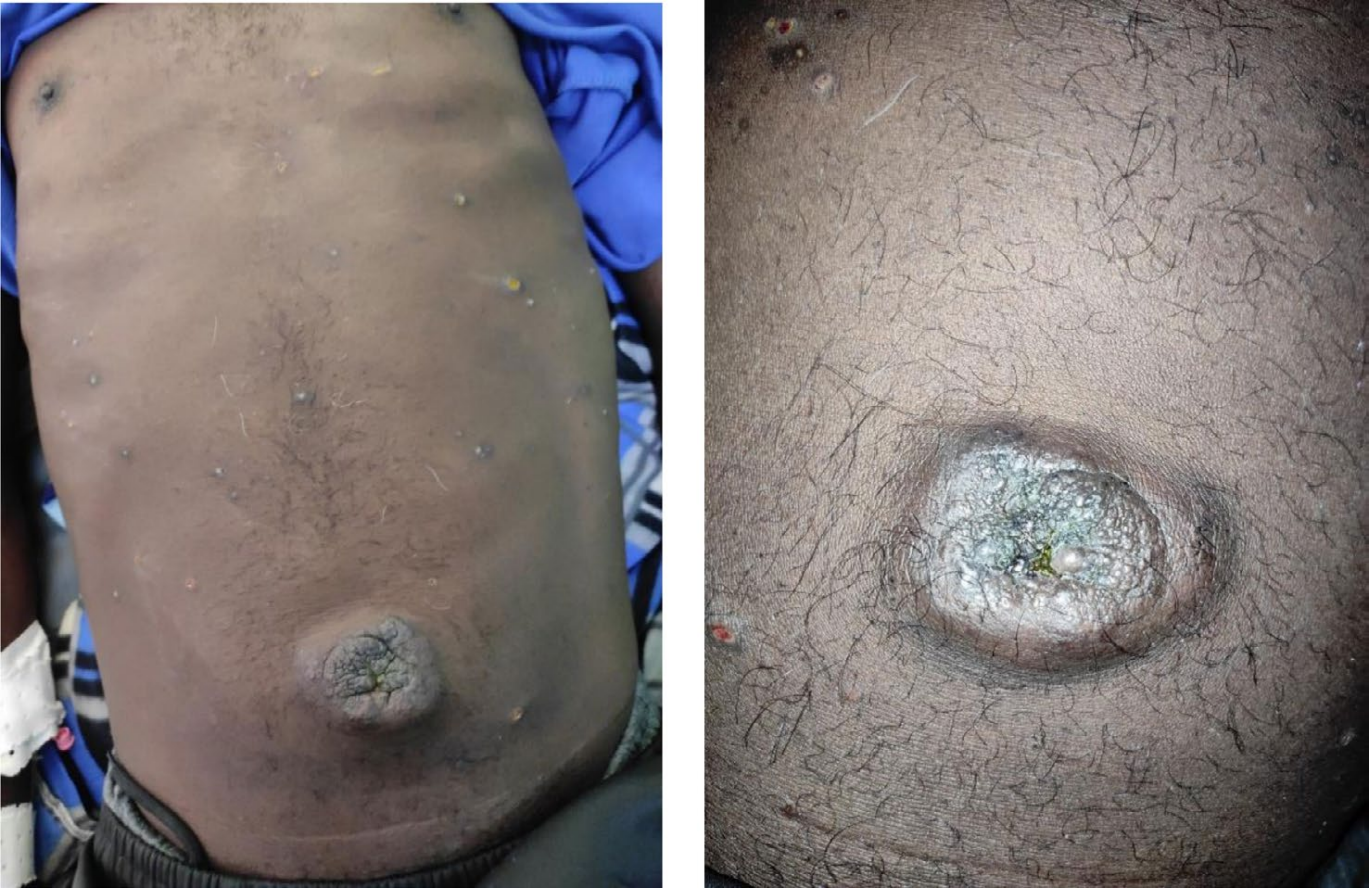
Umbilical nodule as a hyperchromic tumefaction with an irregular surface.

On examination, apart from an altered general condition with a loss of 19% of his initial body weight, he presented with a cholestasis syndrome and an abdomen with multiple scratching lesions. The umbilicus was the site of a hyperchromic, irregular‐surfaced, hard, painful tumefaction measuring 6 cm in long axis (Figure 1). Palpation revealed diffuse abdominal pain with an umbilical mass. There was also medium‐volume ascites.

### 2.2 Paraclinical Examinations

**FIGURE 2:**
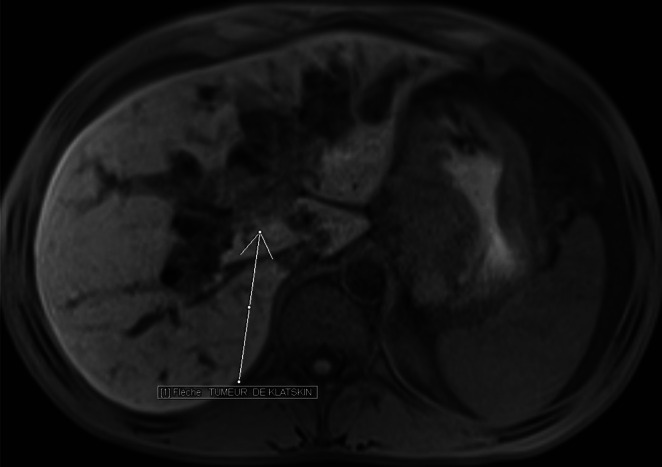
Abdominal MRI T1‐weighted Vibe fs axial section. A voluminous hepatic hilar tissue mass occupying the confluence of the common hepatic duct and the common bile duct is seen in connection with a Bismuth II‐graded extrahepatic cholangiocarcinoma extending to the gallbladder.

**FIGURE 3:**
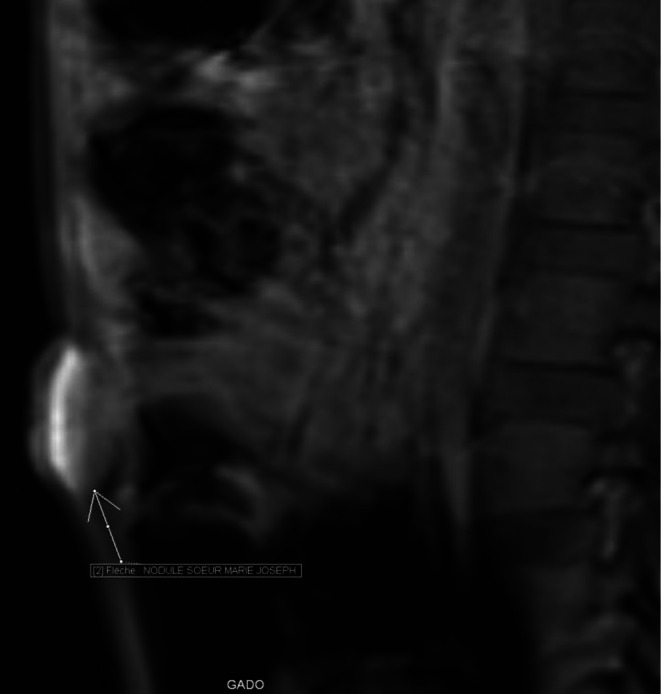
Abdominal MRI T1‐weighted Vibe fs sequence. Sagittal section showing an intensely enhancing umbilical tissue mass after injection of gadolinium contrast medium in connection with the Sister Mary Joseph nodule.

Bili‐MRI (magnetic resonance imaging) revealed a Klatskin's cholangiocarcinoma classified as Bismuth II (Figure 2), as well as a Sister Mary Joseph nodule (Figure 3).

**FIGURE 4:**
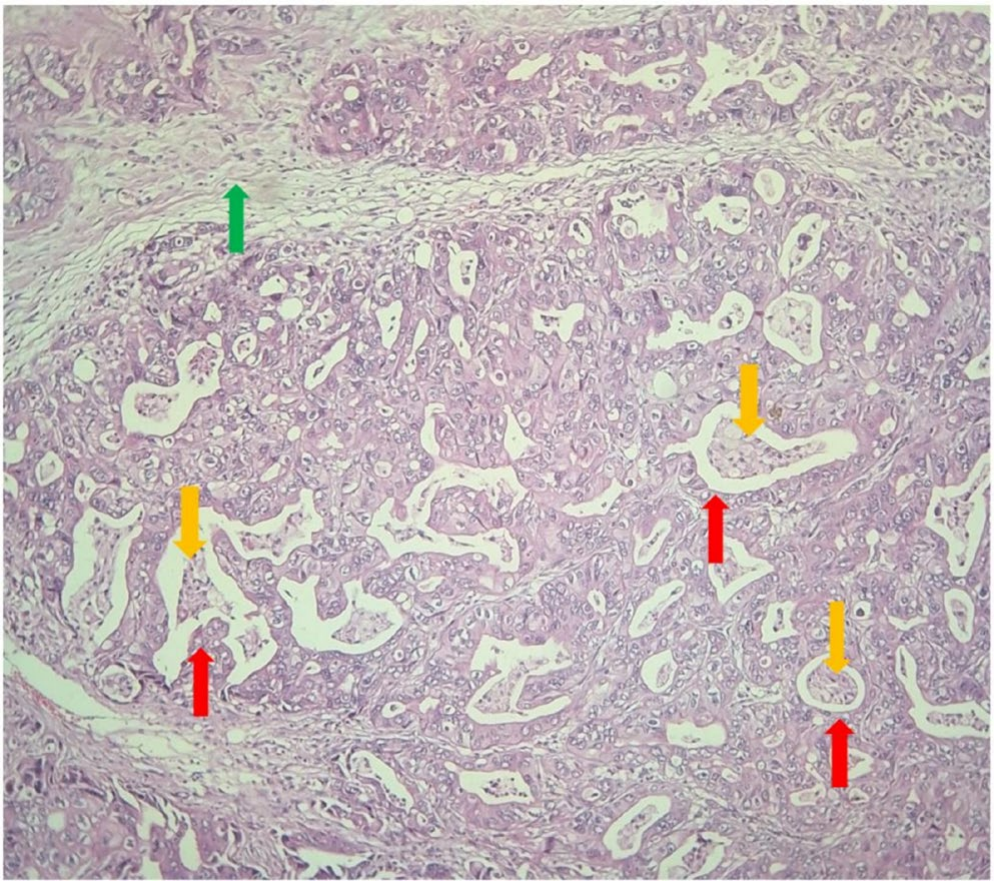
Microphotograph (HE G100): Carcinomatous proliferations (red arrows) centered by comedo‐necroses (yellow arrows). Abundant edematous and inflammatory fibrous stroma (green arrow). This histological image is consistent with a moderately differentiated adenocarcinoma at the umbilical level, which may correspond to a secondary localization of a cholangiocarcinoma.

Pathological examination of the umbilical biopsy showed a moderately differentiated adenocarcinoma, possibly corresponding to a secondary localization of the cholangiocarcinoma (Figure 4).

On biology, total bilirubin was 221 μmol/L, with a conjugated bilirubin/total bilirubin ratio of 91%. His prothrombin level was 87% and anemia was 4.7 g/dL.

### 2.3 Diagnosis

The diagnosis of umbilical cutaneous metastasis of a Klatskin's cholangiocarcinoma classified as Bismuth II was retained.

### 2.4 Outcomes

Given the advanced stage of the disease, the patient's poor general condition, and financial constraints, a supportive care approach was adopted. Management included symptomatic treatment to control pain and pruritus, paracentesis to improve digestive and respiratory comfort, and psychological support. Despite these measures, the patient's condition progressively deteriorated, and he died after 2 months of hospitalization due to sepsis and worsening general status.

## 3 Discussion

From an epidemiological perspective, all authors agree that umbilical cutaneous metastases or Sister Mary Joseph nodules are rare [2, 7, 8]. They may or may not originate from abdominopelvic cancers. In almost a third of cases, the Sister Mary Joseph nodule is the first or only sign of neoplasia [8]. Intra‐abdominal tumors causing Sister Mary Joseph's nodule most often originate in the gastrointestinal tract: stomach, colon, and pancreas in particular. No significant association between gender and these origins has been found. In addition to these organs, women's nodules originate from the ovaries, uterus, and breasts [1, 9]. Rarer localizations have also been reported. Bork et al. [10], Gupta et al. [11], or more recently Khadela et al. [12] reported a case of umbilical metastasis of gallbladder carcinoma. Some authors, such as Ben Kridis et al. [5] and Premkumar et al. [13] reported a case of umbilical metastasis of intrahepatic cholangiocarcinoma. A search on PubMed and ScienceDirect on April 23, 2025, with the terms “Klatskin OR cholangiocarcinoma AND Sister Mary Joseph nodule” yielded roughly 10 results. The etiological investigations of the primary tumor are sometimes complex. In a study carried out in the Netherlands by Hugen et al., the primary tumor was not found in 17.7% of cases [9]. The mechanisms by which tumors metastasize to the umbilicus are not clearly defined. Indeed, there are several hypotheses (5 types) that have been summarized by Balakrishnan et al. [14]:

- Type I: lymphatic spread via the retrograde subserosal lymphatics from axillary, inguinal, and para aortic nodes (common for gynecological, renal tumors);
- Type II: arterial spread through the anastomosis between the inferior epigastric, lateral thoracic, and the internal mammary arteries (common for gynecological tumors);
- Type III: venous spread through (common for gynecological tumors, renal tumors):
  - The anastomotic braches from the axillary veins, internal mammary veins, and the femoral veins;
  - The portal system via the small umbilical veins;
- Type IV: direct extension through the peritoneum (common for gastrointestinal tumors);
- Type V: Through the urachus, remains of omphalomesenteric duct and falciform ligament (common for spread of genitourinary tumors).

A number of hypotheses have been proposed for explaining the spread of these tumors [13]. The precise route of umbilical dissemination remains uncertain in this patient.

The Sister Mary Joseph nodule has no specific clinical manifestation. It may be suspected in the presence of a firm, indurated umbilical tumefaction with irregular margins. The surface may be fissured or ulcero‐necrotic, often painful or pruritic. Size varies, sometimes reaching 10 cm [8]. This description is similar to that of our patient. A serous, bloody, mucoid, or purulent discharge may also be present [8]. The Sister Mary Joseph nodule poses a differential diagnosis problem with other pathologies. These include benign umbilical tumors such as epidermal cysts, epidermal nevi, verrucae vulgaris, dermatofibromas, hypertrophic scars, keloid scars, neurofibromas, and soft fibromas [15]. Other possible diagnoses include an omphalomesenteric cyst, a pyogenic granuloma, endometriosis, an umbilical hernia, or primary umbilical cancer [16, 17]. A medical imaging study can support its diagnosis [17, 18]. On a CT scan, it appears as a retro or peri‐umbilical nodule or mass that is tissue‐dense and enhanced after injection. It has irregular limits and infiltrates the umbilicus [17]. However, a definitive diagnosis is given by an anatomopathological examination of biopsy specimens. Biopsies can be obtained via fine‐needle aspiration. Sister Mary Joseph's nodule indicates an advanced primary tumor. Patients with umbilical skin metastases generally have a poor prognosis. Hugen et al. reported a median survival of 7.9 months [9]. In Ivory Coast, Turquin et al. described two cases of umbilical metastases from visceral tumors, with the patients dying 7 days and 3 months after hospitalization, respectively [2]. Nevertheless, longer survival times have also been reported in other cases [19, 20, 21]. Depending on the stage of the cancer and the patient's condition, different therapeutic approaches can be undertaken. Radiotherapy may also be used [7].

## 4 Conclusion

Sister Mary Joseph nodule is a rare clinical manifestation. This nodule has no specific clinical signs. Any change in the appearance of the umbilicus should be taken into account, hence the importance of a thorough clinical examination. Its onset is late and is associated with a poor prognosis. Our patient died 2 months after diagnosis.

## Author Contributions

**Lawagoulé Joseph Emile Ky:** conceptualization, methodology, supervision, validation, visualization, writing – original draft. **Eric Hervé Mourfou:** visualization, writing – original draft. **Abdoul Fatao Maïga:** visualization, writing – original draft. **Daouda Sangaré:** visualization, writing – original draft. **Damien Marcel Traoré:** visualization, writing – original draft. **Saïdou Santi:** visualization, writing – original draft. **Madina Koanda:** visualization, writing – original draft. **Alimata Sanfo:** visualization, writing – original draft. **Delphine Napon/Zongo:** validation. **Aboubacar Coulibaly:** supervision. **Kounpiélimé Sosthène Somda:** supervision. **Arsène Roger Sombié:** supervision.

## Ethics Statement

The authors have nothing to report.

## Consent

Authors obtained written patient consent in accordance with the journal's patient consent policy, and that we have added a patient consent statement asserting this at the bottom of the manuscript's title page. We will retain the original written consent form and provide it to the Publisher if requested.

## Conflicts of Interest

The authors declare no conflicts of interest.

## Data Availability

The data that support the findings of this study are avail able from the corresponding author upon reasonable request.
